# A next generation approach to species delimitation reveals the role of hybridization in a cryptic species complex of corals

**DOI:** 10.1186/s12862-019-1427-y

**Published:** 2019-06-06

**Authors:** Andrea M. Quattrini, Tiana Wu, Keryea Soong, Ming-Shiou Jeng, Yehuda Benayahu, Catherine S. McFadden

**Affiliations:** 1 Biology Department, Harvey Mudd College, 1250 N. Dartmouth Ave, Claremont, CA 91711 USA; 2 Institute of Marine Biology, National Sun Yat-sen University, Kaohsiung, Taiwan; 3 Biodiversity Research Center, Academia Sinica, Taipei, Taiwan; 4 School of Zoology, George S. Wise Faculty of Life Sciences, Tel Aviv University, 69978 Ramat Aviv, Israel

**Keywords:** Hybridization, RADSeq, Phylogenetics, Coral reefs, Taxonomy, Anthozoa, Octocorallia

## Abstract

**Background:**

Our ability to investigate processes shaping the evolutionary diversification of corals (Cnidaria: Anthozoa) is limited by a lack of understanding of species boundaries. Discerning species of corals has been challenging due to a multitude of factors, including homoplasious and plastic morphological characters and the use of molecular markers that are either not informative or have not completely sorted. Hybridization can also blur species boundaries by leading to incongruence between morphology and genetics. We used traditional DNA barcoding and restriction-site associated DNA sequencing combined with coalescence-based and allele-frequency methods to elucidate species boundaries and simultaneously examine the potential role of hybridization in a speciose genus of octocoral, *Sinularia.*

**Results:**

Species delimitations using two widely used DNA barcode markers, *mtMutS* and 28S rDNA, were incongruent with one another and with the morphospecies identifications. When *mtMutS* and *28S* were concatenated, a 0.3% genetic distance threshold delimited the majority of morphospecies. In contrast, 12 of the 15 examined morphospecies formed well-supported monophyletic clades in both concatenated RAxML phylogenies and SNAPP species trees of > 6000 RADSeq loci. DAPC and Structure analyses also supported morphospecies assignments, but indicated the potential for two additional cryptic species. Three morphologically distinct species pairs could not, however, be distinguished genetically. ABBA-BABA tests demonstrated significant admixture between some of those species, suggesting that hybridization may confound species delimitation in *Sinularia.*

**Conclusions:**

A genomic approach can help to guide species delimitation while simultaneously elucidating the processes generating coral diversity. Results support the hypothesis that hybridization is an important mechanism in the evolution of Anthozoa, including octocorals, and future research should examine the contribution of this mechanism in generating diversity across the coral tree of life.

**Electronic supplementary material:**

The online version of this article (10.1186/s12862-019-1427-y) contains supplementary material, which is available to authorized users.

## Background

The ability to delimit species is fundamental to the accurate assessment of biodiversity and biogeography, information that is essential for studying community ecology as well as for implementing conservation policies. Yet this task is not trivial, as species are often difficult to discriminate for a multitude of reasons. Morphological traits have traditionally been used in classical taxonomy; however, use of characters that might not be diagnostic or are homoplasious can confound the interpretation of species boundaries. Cryptic species, particularly those that occur in sympatry, and species that have arisen via hybridization and introgression are often challenging to discriminate without genetic, ecological or behavioral data. DNA barcoding of mitochondrial genes has proven useful in many species groups [46, 47], but incomplete lineage sorting and past hybridization events complicate species delimitation based on mitochondrial data alone, particularly in recently diverged taxa [49, 53, 73]. In addition, mitochondrial markers reflect the history of maternal lineages, which are often incongruent with the species’ history [18, 89]. The increased resolution of genomic data can potentially disentangle some of these issues, facilitating species delimitation while simultaneously furthering our understanding of processes that generate biodiversity [103]. Moreover, such an approach may also provide a better evaluation of morphological traits and insights into their congruence with genetic data.

In sessile marine invertebrates, such as corals, congeners often occur in sympatry and occupy similar ecological niches and reef zones. These ecological characteristics combined with reproductive modes may lead to increased rates of hybridization among close relatives. Broadcast-spawning species that occur in sympatry often participate in synchronous, mass-spawning reproduction events [2, 44, 55, 88]. Unless there are prezygotic mechanisms to reproductive isolation, such as gametic incompatibility or asynchronous spawning times, there may be numerous opportunities for hybridization to occur [109, 113, 114]. In fact, laboratory crossings of sympatric congeners have produced viable hybrid offspring in several species [101, 113]. Hybridization followed by reticulate evolution has been suggested to be an important mechanism generating the species diversity observed in some groups of corals [17, 20, 39, 45, 69, 74, 87, 108, 109, 114]. Vollmer and Palumbi [112], however, suggested that hybridization could yield distinct, new morphotypes that may be reproductively inviable or subject to hybrid breakdown. It is clear that further investigation is needed to determine the potential contributions of hybridization to speciation and morphological innovation in corals.

One particularly speciose group of octocorals (Cnidaria: Anthozoa: Octocorallia) is the genus *Sinularia* May, 1898. This zooxanthellate genus includes approximately 175 valid species [115], 47 of them described just in the last 25 years. They are diverse and abundant throughout the Indo-Pacific, and biodiversity surveys of shallow-water coral reef communities typically report more than 15 co-occurring species of *Sinularia*, with as many as 38 species recorded at some locations [67, 75, 76]. *Sinularia* species are typically most abundant on reef flats and shallow slopes, where single- or multi-species assemblages may dominate the reef substrate [4, 5, 21, 31, 105]. *Sinularia* can also play an active role in reef biogenesis through deposition of spiculite formed by the cementation of layers of calcitic sclerites [52, 98]. In addition, many *Sinularia* species produce secondary metabolites used for allelopathy and predator deterrence [99, 100, 106, 116], making the genus a rich and diverse source of bioactive natural products (e.g., Blunt et al., [9]).

Because of the dominance and importance of *Sinularia* species across a wide depth gradient [97] as well as their susceptibility to bleaching-induced mortality [12, 32, 41, 68], it is of great interest to better understand their ecology and function on the reefs. Ecological studies, however, are often hampered by the uncertainty of species identifications [70]. Classical taxonomy of *Sinularia* species is based primarily on morphological features of the colony and the shape and dimension of sclerites (microscopic calcitic skeletal elements) found in different parts of the colony [33, 70, 111]. Separation of species using these characters can be subjective, as the complex morphologies of both colonies and sclerites are rarely quantified [1, 14]. There is also a potential contribution of environmental plasticity to the morphological variation observed, as has been documented in other octocorals [59, 92, 93].

The application of molecular systematic and DNA barcoding approaches to the study of species boundaries in *Sinularia* have been only partially successful [70]. While molecular approaches have revealed that some well-known morphospecies comprise cryptic species complexes [77, 78], it is also the case that numerous morphologically distinct *Sinularia* species share identical haplotypes at barcoding loci [8, 70, 72]. Because mitochondrial genes evolve slowly in Anthozoa [50, 96], these markers often simply lack the resolution to distinguish recently diverged species [71, 72]. As a result, it is often not possible to conclude with certainty whether morphologically distinct individuals that share identical DNA barcodes represent different octocoral species or morphological variants of a single species. In addition, the reported ability of some species of *Sinularia* to hybridize in the laboratory [101], raises the possibility that naturally occurring hybridization events could contribute to the observed morphological diversity of this genus, as has been suggested for stony corals [87]. The true identity of some *Sinularia* species remains uncertain, and our ability to explore the evolutionary processes leading to diversification of this hyperdiverse lineage are limited by a lack of understanding of species boundaries. In fact, we have a limited understanding of species boundaries for numerous groups of recently diverged corals (e.g., [53, 73, 107]).

Octocoral biodiversity surveys conducted recently at Dongsha Atoll, South China Sea, Taiwan, recorded 27 nominal morphospecies of *Sinularia* inhabiting the reef slope down to a depth of 20 m [8], most of them belonging to the speciose clades “4” and “5C” [70]. These two clades include several subclades each characterized by a different suite of morphological characters whose diagnosis is quite confusing [70]. While most of these morphospecies could be distinguished using a character-based mitochondrial gene barcode (*mtMutS*), five distinct morphospecies in clade 5C shared identical haplotypes, and several morphospecies in both clades were represented by more than one haplotype [8]. These morphospecies exemplify a problem common to many corals and raise the following questions: (1) do the observed morphological differences reflect boundaries between species whose mitochondrial haplotypes have not yet diverged or coalesced, or do these differences reflect intraspecific variation? and (2) might the sharing of mitochondrial haplotypes among distinct morphotypes reflect ongoing or past hybridization events?

To further explore these questions and to elucidate species boundaries in the *Sinularia* that co-occur on Dongsha Atoll, we have (1) sequenced an additional, nuclear marker (*28S rDNA*) that has been shown to be comparable to *mtMutS* as a species-specific barcode for *Sinularia* and other octocoral taxa [72]; and (2) sequenced restriction-site associated DNA (RADseq) to identify SNPs for multilocus species delimitation analyses using allele-frequency and coalescence-based approaches. Expanding upon a recent study of co-occurring *Sinularia* species at Dongsha Atoll [8], we validate morphospecies identifications using a genomic approach, and provide insight into the possible role of hybridization in the evolution of the genus.

## Results

### Species delimitation using DNA barcodes

**Fig. 1:**
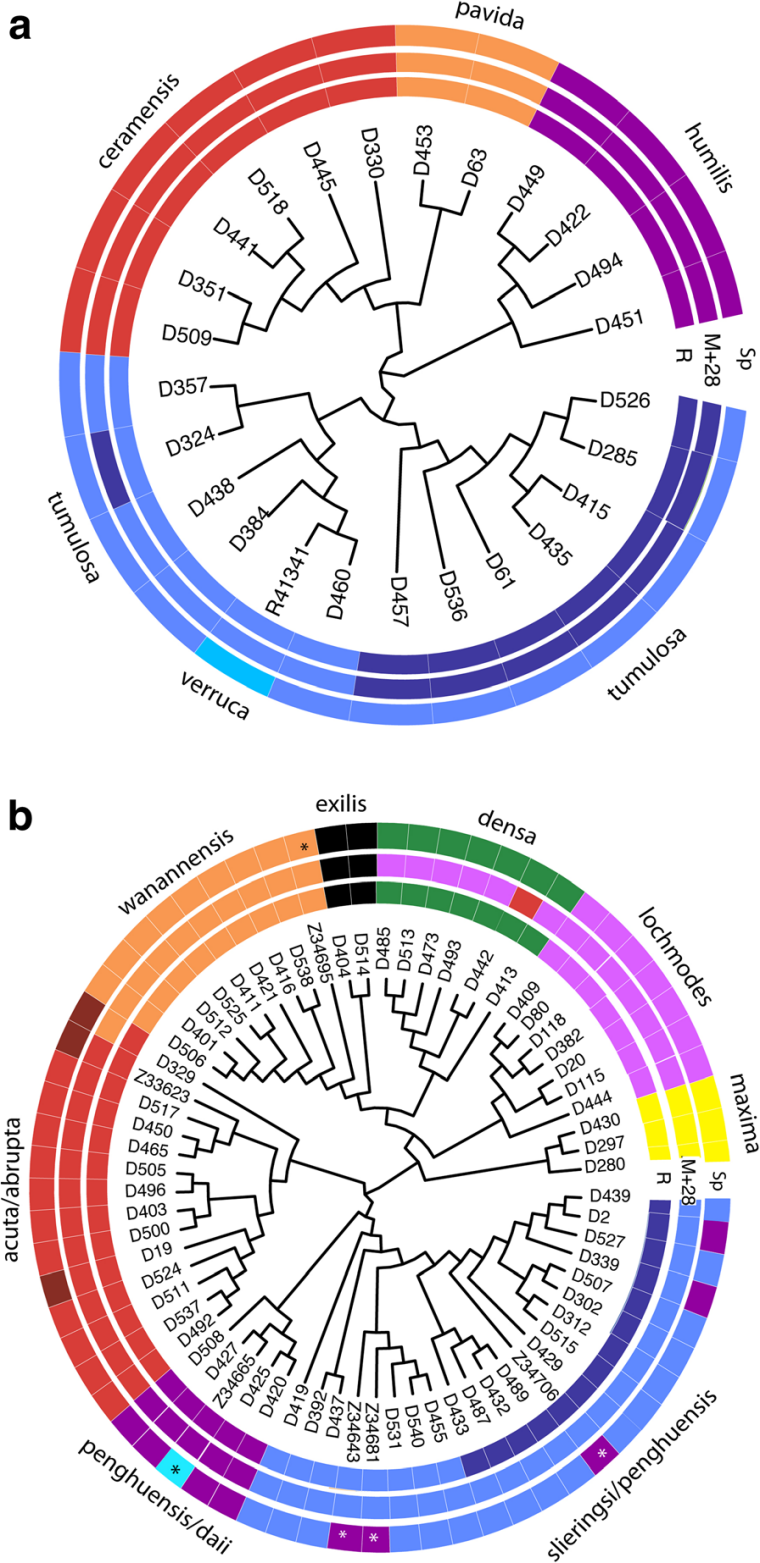
Maximum likelihood cladograms for Dongsha Atoll *Sinularia* **a** clade 4 and **b** clade 5C. Each colored cell denotes an individual’s species assignment based on RAD data (R), a morphospecies assignment (Sp), or a molecular operational taxonomic unit (MOTU) based on *mtMutS* and 28S rDNA (M + 28). Colors match RAD clades in Figs. 3 and 4. * = holotype or paratype. Morphospecies names are also included. See also Additional file 4 for clade and MOTU assignments

Neither the *mtMutS* (735 bp) nor the *28S rDNA* (764 bp) barcoding marker delimited all morphospecies of *Sinularia* (Table 1) when considered separately (Additional file 1: Figure S1, Additional file 4). Phylogenetic relationships among morphospecies were poorly resolved with low support values and few reciprocally monophyletic groups, especially in clade 5C (Additional file 1: Figure S1). Based on a 0.3% genetic distance threshold, the *mtMutS* barcode identified six molecular operational taxonomic units (MOTUs) among the four morphospecies belonging to clade 4, splitting *S. tumulosa* and *S. ceramensis* into two MOTUs each (Additional file 1: Figure S1). In contrast, *28S rDNA* (0.3% threshold) delimited only four MOTUs within clade 4, each of them congruent with morphospecies identifications. The only exception was *S. verruca* (R41341) from Palau, included as a taxonomic reference, whose *mtMutS* and *28S* sequences were identical to those of *S. tumulosa*. When the two barcode markers were concatenated, application of a 0.3% genetic distance threshold delimited five species in clade 4: *S. humilis*, *S. pavida* and *S. ceramensis* were delimited clearly, but individuals of *S. tumulosa* were split among two putative species, one of which included *S. verruca* (Fig. 1a).

**Table 1:** Morphospecies of *Sinularia* included in RADseq analysis

Species	Authority	Clade	N	#Sites	Depth (m)
*S. abrupta*	Tixier-Durivault,1970	5C	3	2	8–13
*S. acuta*	Manuputty & Ofwegen, 2007	5C	11	6	4–14
*S. ceramensis*	Verseveldt, 1977	4D	6	5	3–13
*S. daii* ^a^	Benayahu & Ofwegen, 2011	5C	1	-	---
*S. densa*	(Whitelegge, 1897)	5C	6	4	3–14
*S. exilis*	Tixier-Durivault, 1970	5C	2	2	5–14
*S. humilis*	Ofwegen, 2008	4B	4	3	3–21
*S. lochmodes*	Kolonko, 1926	5C	8	6	4–14
*S. maxima*	Verseveldt, 1971	5C	3	2	3–17
*S. pavida*	Tixier-Durivault, 1970	4D	2	2	4–5
*S. penghuensis*	Ofwegen 2012	5C	8	3	3–21
*S. slieringsi*	Ofwegen & Vennam, 1994	5C	17	6	3–21
*S. tumulosa*	Ofwegen, 2008	4D	12	8	3–17
*S. verruca* ^a^	Ofwegen, 2008	4D	1	-	---
*S. wanannensis*	Ofwegen 2012	5C	9	5	5–21

Clade corresponds to designations in McFadden et al. [70]. N: number of specimens sequenced. #Sites: number of different dive sites at Dongsha Atoll at which a species was collected

^a^reference species not collected at Dongsha

Among the clade 5C morphospecies, *mtMutS* delineated eight MOTUs (Additional file 1: Figure S1). With just two exceptions (D442, Z34695), *mtMutS* differentiated *S. maxima*, *S. wanannensis*, *S. lochmodes* and *S. densa* from all other morphospecies (Additional file 1: Figure S1). A majority of the colonies identified as *S. penghuensis* and *S. slieringsi*, both individuals of *S. exilis*, and seven of eleven *S. acuta*, however, belonged to a single MOTU, while the remaining four individuals of *S. acuta* were assigned to a separate MOTU. Two individuals with divergent haplotypes (*S. penghuensis* D002 and *S. slieringsi* D439) were each assigned to unique MOTUs. In contrast to *mtMutS*, *28S* delineated only four MOTUs within clade 5C (Additional file 1: Figure S1). *S. penghuensis* and *S. slieringsi*, which were not separated using *mtMutS*, were separated into two distinct MOTUs; four of the *S. penghuensis* colonies belonged to a MOTU that was well separated from all others and also included *S. daii*, while the other five—including the holotype (Z34706) and both paratypes—shared identical genotypes with *S. slieringsi*. A third MOTU included *S. acuta*, *S. densa* and *S. lochmodes* along with *S. abrupta* and one *S. exilis*. The fourth MOTU included all individuals of *S. maxima* and *S. wanannensis* plus the second *S. exilis*.

When *mtMutS* and *28S* were concatenated, a 0.3% genetic distance threshold delimited seven MOTUs in clade 5C, five of them largely congruent with morphospecies definitions (*S. maxima*, *S. exilis*, *S. acuta*, *S. wanannensis* and *S. slieringsi*) (Fig. 1b; Additional file 1: Figure S2). *S. penghuensis* was divided among two MOTUs: five individuals (including all three type specimens) belonged to the same MOTU as *S. slieringsi*, while the other four were placed in a MOTU with *S. daii*. *S. lochmodes* and all but one individual of *S. densa* were lumped together in a seventh MOTU. Several individuals did not fall into a MOTU with others belonging to the same morphospecies: two individuals of *S. abrupta* (D119, Z33623) and one *S. densa* (D442) were not distinguished from *S. acuta,* while the third *S. abrupta* (D329) was lumped with *S. wanannensis* (Fig. 1b). Two of the individuals of *S. abrupta* showed incongruence between *mtMutS* and *28S* genotypes: D329 had a *mtMutS* sequence matching *S. wanannensis* but a *28S* sequence matching *S. acuta*, while Z33623 had a *mtMutS* sequence matching *S. acuta* but its *28S* matched *S. densa*. In contrast, D019 was identical to *S. acuta* at both loci. The holotype of *S. wanannensis* from Penghu (Z34695) also had incongruent barcode genotypes; its *28S* sequence was consistent with other individuals of that species, but it shared a *mtMutS* haplotype with *S. penghuensis* and *S. slieringsi* (Additional file 1: Figure S1).

### RADSeq data statistics

A total of 289,373,374 reads were obtained for 95 *Sinularia* samples. After trimming in both Stacks and pyRAD, 86% of reads were retained (247,873,622). The mean number of reads per individual was 2,609,196 ± 627,314. For each clade, the number of loci and the number of SNPs obtained increased considerably when the number of shared heterozygous sites (*p)* was increased and both the clustering threshold (*c*) and individual occupancy per locus (*m*) were decreased (Table 2). Notably, a substantial increase in both the number of loci and SNPs obtained occurred when *p* was set to 0.25 at a clustering threshold (*c*) of 0.85. The number of loci obtained ranged from 73 to 28,179 for clade 4 and 115 to 23,946 for clade 5C depending upon parameters used in pyRAD analyses (Table 2). The number of variable SNPs obtained ranged from 382 to 251,615 for clade 4 and 885 to 329,837 for clade 5C (Table 2).

**Table 2:** Dongsha Atoll *Sinularia*. Loci and SNP summary statistics of pyRAD simulations at two different clustering thresholds (*c*), three different levels of taxon occupancy per locus (*m),* and two different levels of shared polymorphic sites *(p)*. Minimum and maximum loci obtained for one individual are indicated as well as total loci obtained across all individuals. Total number of variable SNPs (Var), parsimony informative SNPs (PI), and unlinked bi-allelic SNPs (BI) are also included

(m)	(c)	Number of Loci						Number of SNPs					
		Min	Max	Total	Min	Max	Total	Var.	PI	BI	Var.	PI	BI
Clade 4			*p10*			*p25*			*p10*			*p25*	
1.0	0.90	73	73	73	154	154	154	382	78	63	1,358	617	144
	0.85	93	93	93	185	185	185	563	255	83	1,675	778	175
0.75	0.90	1,570	2,391	2,552	3,139	5,331	5,515	17,690	10,505	2,448	48,301	28,211	5,411
	0.85	1,869	2,869	2,958	3,791	6,113	6,343	22,936	14,026	2,851	60,232	36,467	6,236
0.50	0.90	3,391	9,742	10,813	6,923	24,178	26,861	70,048	38,239	10,490	214,870	121,983	26,532
	0.85	4,347	10,341	11,540	8,693	25,221	28,179	86,607	49,558	11,225	251,615	147,779	27,860
Clade 5C													
1.0	0.90	115	115	115	143	143	143	885	212	104	1,222	356	132
	0.85	123	123	123	154	154	154	908	229	109	1,325	385	140
0.75	0.90	2,500	4,174	4,306	4,060	6,675	7,083	52,325	31,844	4,276	91,810	57,263	7,051
	0.85	2,781	4,793	4,968	4,491	7,771	8,060	62,057	38,113	4,930	106,548	66,726	8,022
0.50	0.90	6,210	11,587	13,189	9,439	20,926	23,946	171,091	104614	13141	329,837	205,990	23,898
	0.85	5,381	11,697	13,281	9,329	20,582	23,484	175,558	108,258	13,225	327,211	205,557	23,428

### Phylogenetic inference and species delimitation

**Fig. 2:**
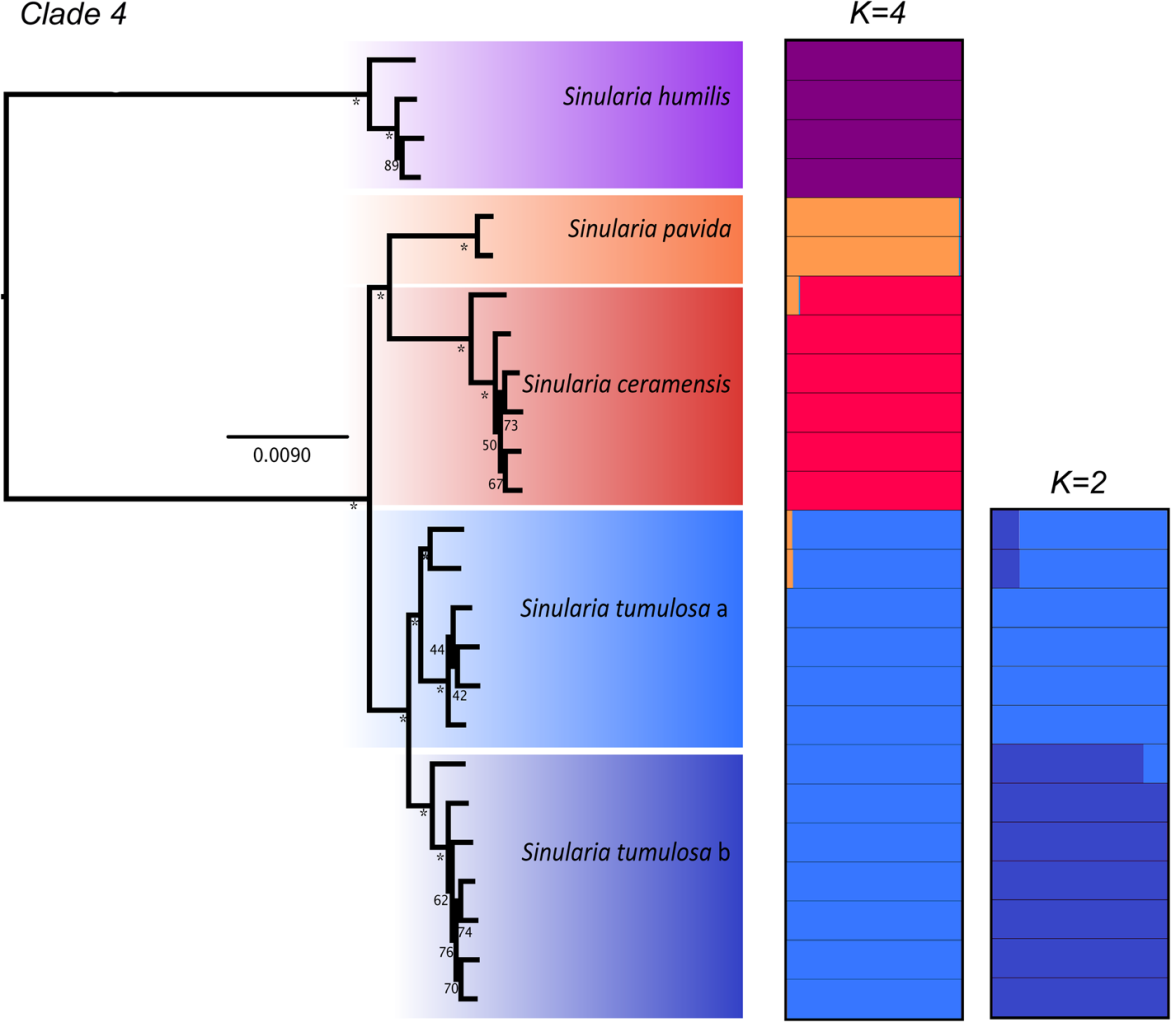
Maximum likelihood phylogeny of Dongsha Atoll *Sinularia* clade 4 constructed using RAxML rapid bootstrapping (200 b.s. replicates) on the concatenated c 0.85, m 0.75, p 0.25 locus dataset. * denotes 100% b.s. support. Distruct plots are included and show the probability of individual membership into different *K* clusters (*K* = 4 for clade 4 and *K* = 2 for the *S. tumulosa* group). Colors denote different species. *S. tumulosa* is designated as ‘a’ and ‘b’ as results strongly suggest this clade consists of two species

**Fig. 3:**
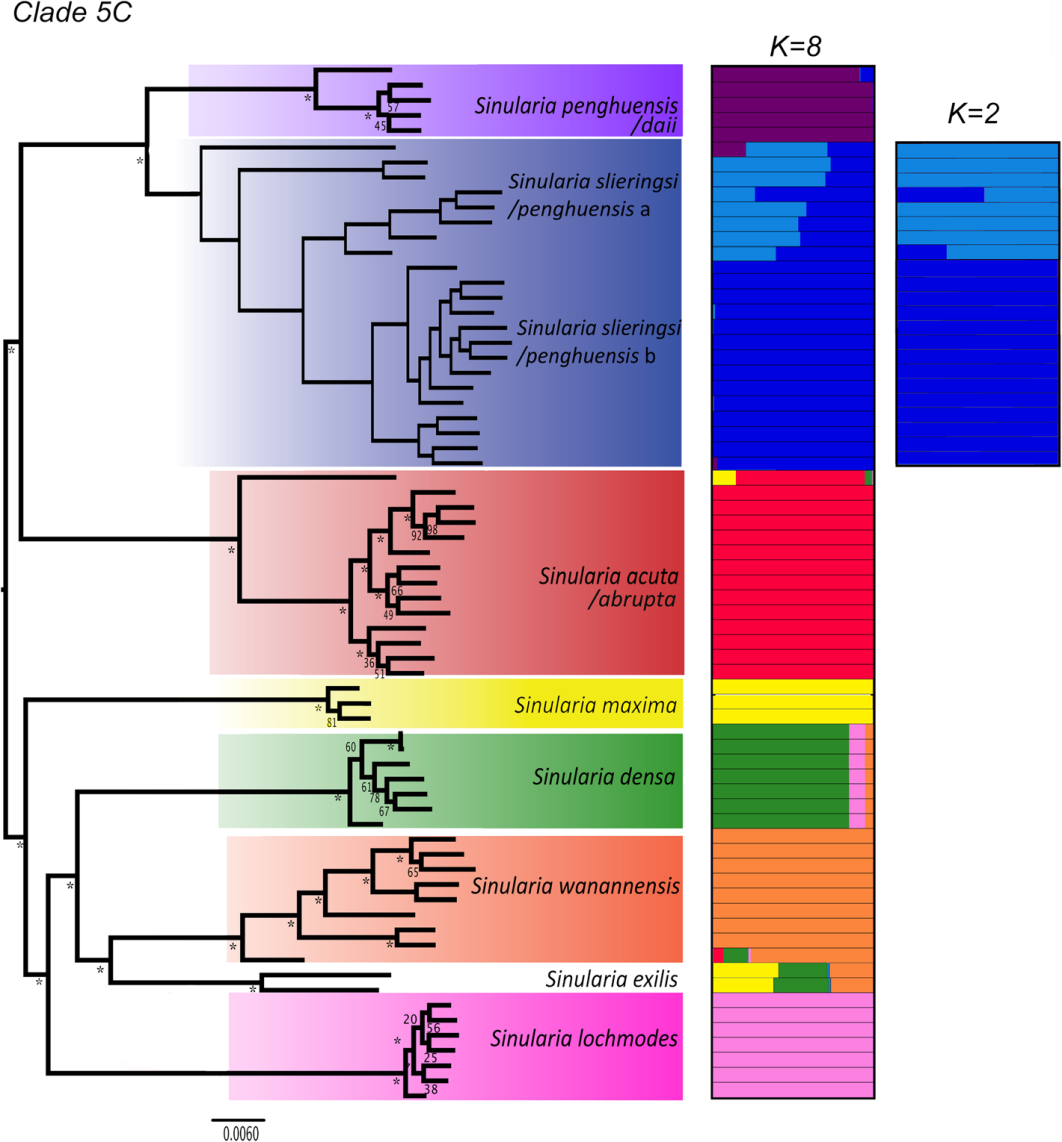
Maximum likelihood phylogeny of Dongsha Atoll *Sinularia* clade 5C constructed using RAxML rapid bootstrapping (200 b.s. replicates) on the concatenated c 0.85, m 0.75, p 0.25 locus dataset. * denotes 100% b.s. support. Distruct plots are included and show the probability of membership into different *K* clusters (*K* = 8 for clade 5C, and *K* = 2 for the *S*. *slieringsi/penghuensis* group). Colors denote different species. *S. slieringsi/penghuensis* is designated as ‘a’ and ‘b’ as results suggest this clade may consist of two species

**Fig. 4:**
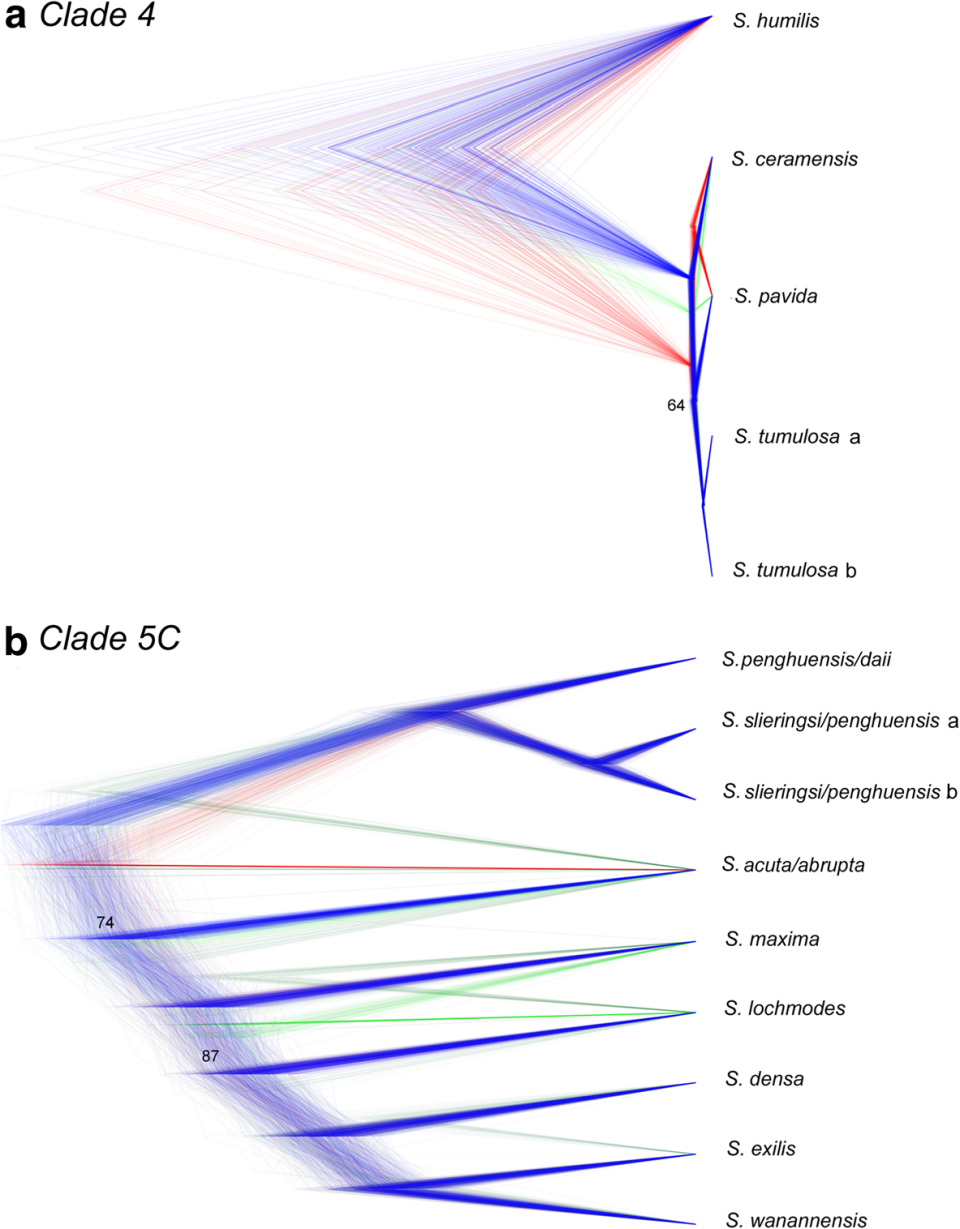
Species trees of Dongsha Atoll *Sinularia* clades 4 and 5C*.* Cloudograms illustrate the best species delimitation models (DAPC+ 1) for both clades inferred from bi-allelic SNP data [**a** clade 4: 6,236 SNPs and **b** clade 5C: 8,022 SNPs); *m*0.75 datasets] using SNAPP species tree analyses. The maximum clade credibility tree and congruent trees are in blue. Trees with different topologies are in red and green. Posterior probabilities at internal nodes > 95% unless indicated

Similar to the combined analysis of concatenated *mtMutS* and *28S rDNA* barcodes (Fig. 1, Additional file 1: Figures S1 and S2), a majority of the identified morphospecies formed well-supported monophyletic clades in both clade 4 and 5C phylogenies constructed with the *c* 0.85, *p* 0.25, and *m* 0.75 RADSeq datasets (clade 4: 6,343 loci, clade 5C: 8,060 loci; Figs. 2, 3). The maximum clade credibility species trees produced from SNP data in the SNAPP analyses were largely congruent with the RAxML trees generated from concatenated data (Figs. 2, 3, 4). However, in clade 4, *S. pavida* and *S. ceramensis* were reciprocally monophyletic in the ML tree (Fig. 2) but not in the maximum clade credibility SNAPP species tree (Fig. 4a), although this relationship was evident in 30% of the alternative SNAPP tree topologies (in red, Fig. 4a). In clade 5C, *S. acuta* was sister to *S. penghuensis* and *S. slieringsi* in the ML tree (Fig. 3), but not in the maximum clade credibility species tree (Fig. 4b), although this relationship was evident in 25% of the alternative SNAPP tree topologies (in red and green, Fig. 4b). For clade 4, 35% of the SNAPP topologies obtained were alternative to the maximum clade tree, and for clade 5C 37% of trees had different topologies compared to the maximum clade credibility tree (Fig. 4).

### Clade 4

**Fig. 5:**
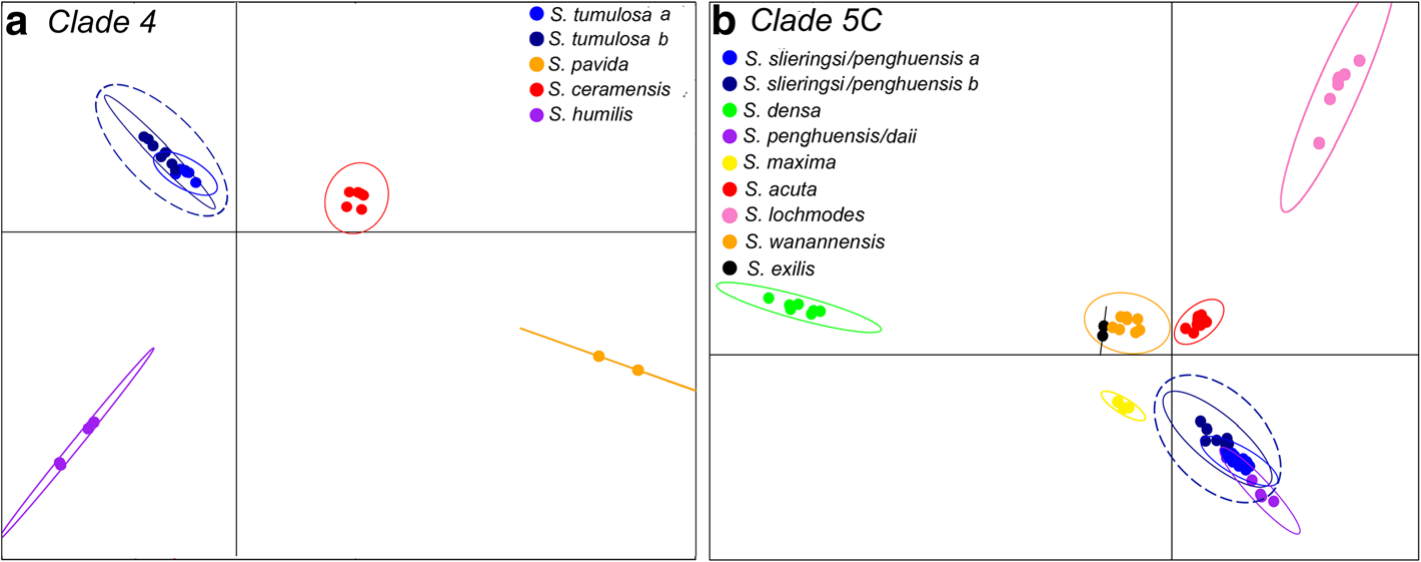
Discriminant analysis of principal components (DAPC) plots for Dongsha Atoll *Sinularia* **a** clade 4 and **b** clade 5C. Genetic clusters representing different morphospecies are color coded to match the phylogenetic trees in Figs. 2, 3 and 4. Species (*S. tumulosa* in (**a**) and *S. slieringsi/penghuensis* in (**b**) encircled in dotted lines) that were suggested to be further divided into two species by Bayes Factor Determination are also denoted

DAPC and Structure analyses supported the currently defined morphospecies in *Sinularia* clade 4 (*S. ceramensis*, *S. humilis, S. pavida,* and *S. tumulosa*), and agreed with the four MOTUs identified by the *28S rDNA* barcoding marker (Additional file 1: Figure S1). Consistent with the barcoding results, *S. verruca* was genetically indistinguishable from *S. tumulosa*. The optimal number of *K* clusters suggested by the DAPC analyses was four (BIC = 120.4, Additional file 1: Figure S3), and the DAPC plot revealed no overlap among these four distinct clusters (Fig. 5a). Further support for group assignment can be seen in the assignment plots, as all individuals were successfully re-assigned into their respective clusters (Additional file 1: Figure S4). In addition, the Distruct plot clearly illustrated little to no admixture among these four species (Fig. 2). Upon further Structure analysis, little to no admixture was also revealed between two sub-clades of *S. tumulosa* (Fig. 2), suggesting that *S. tumulosa* might consist of two species. Other methods also support this result. First, following a one-species model (MLE = -1119), DAPC+ 1 was the second most likely (MLE = -1291) species model according to BFD* analyses (Table 3). The DAPC+ 1 model included species denoted by DAPC, plus two sub-clades of *S. tumulosa*. Second, most of the individuals of these two sub-clades formed two separate groupings in the DAPC plot, although there was some overlap among individuals (Fig. 5a). Finally, *S. tumulosa* was divided into two well-supported, reciprocally monophyletic clades (sp. a and b) in both concatenated and species tree phylogenies, which match the *mtMutS* and combined *mtMutS + 28S* results (Figs. 1a, 2 and 4a; Additional files 1: Figures S1 and S2). It is possible that these represent two cryptic species.

**Table 3:** SNAPP results for different species delimitation models for Dongsha Atoll *Sinularia* clades 4 and 5C

Model	MLE	BF	Rank
*Clade 4*			
ONESP	− 1119	--	1
TWOSPP	− 1450	−662	6
THREESPP	− 1327	− 416	5
mtMutS	− 1300	−362	3
DAPC/28 s	− 1310	−382	4
DAPC+ 1	−1291	− 344	2
*Clade 5C*			
ONESP	−1298	--	1
TWOSPP	− 1660	− 724	7
FOURSPP	− 1594	− 592	4
mtMutS	− 1625	− 654	6
28 s	−1607	− 618	5
DAPC	− 1544	− 492	3
DAPC+ 1	−1543	−490	2

Rank of most likely species model based on Bayes Factor delimitation is indicated

*MLE* Marginal Likelihood Estimate, *BF* Bayes Factor

**Fig. 6:**
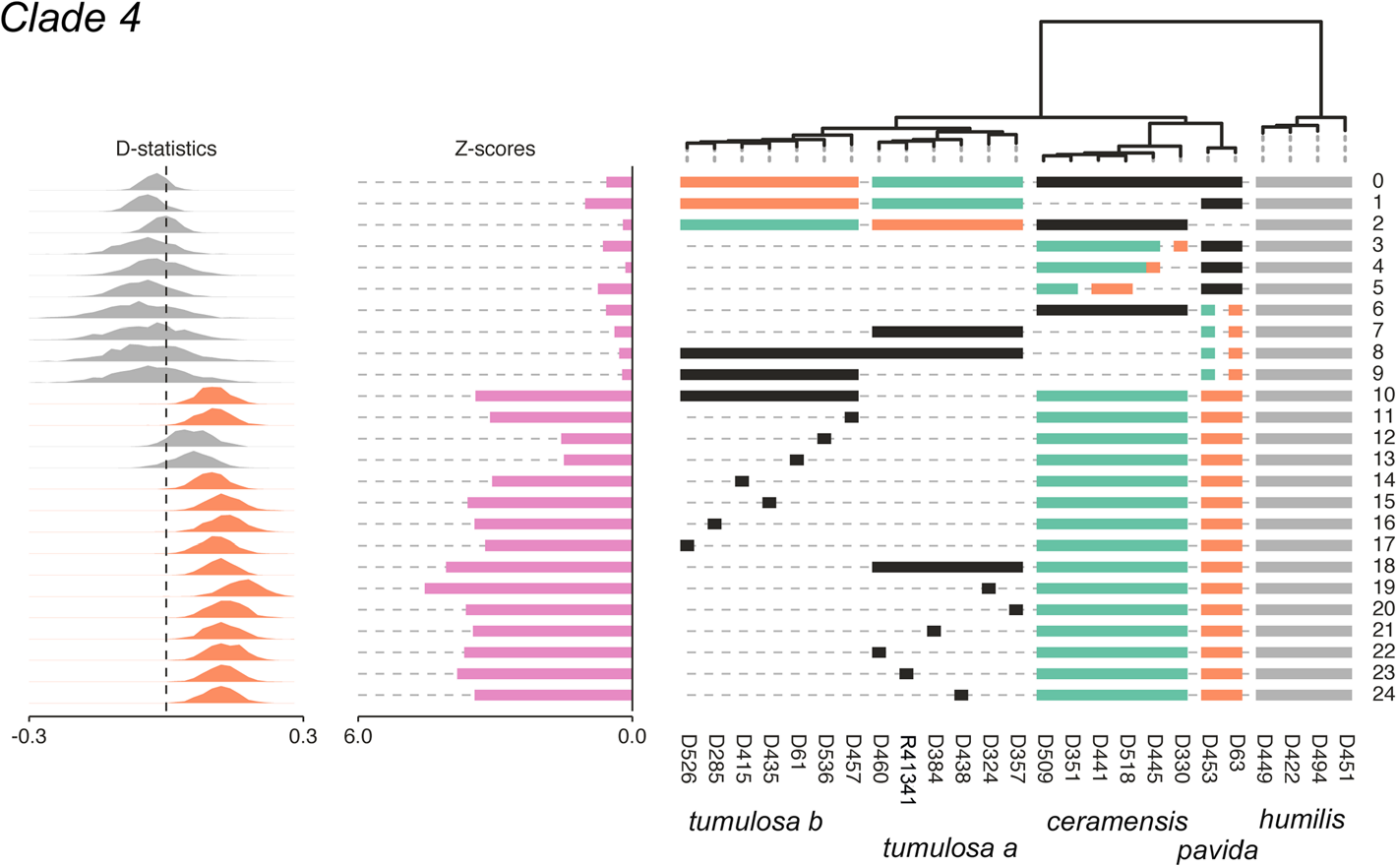
D-statistic tests for admixture in Dongsha Atoll *Sinularia* clade 4. Test numbers are listed on the right for each 4-taxon test (((p1, p2), p3), p4). Horizontal bars below the tips of the tree indicate which taxa were included in each test. *S. humilis* was set as the outgroup for all tests (indicated by gray bars). Tests are configured to ask whether P3 (black bars) shares more derived SNPs with lineage P1 (green bars) relative to P2 (orange bars). As illustrated to the left, Z scores are bar plots and D-statistics are histograms. Histograms are green for significant gene flow between P1 and P3 (BABA) and orange for significant gene flow between P2 and P3 (ABBA). D-statistics that were not significant are gray. Significance was assessed at an alpha level of 3.0 (i.e., when D deviates > 3.0 standard deviations from zero)

Twenty-four separate ABBA-BABA tests were performed on clade 4 (Fig. 6, Additional file 2). The average number of loci shared across taxa in each test was 3313 ± 556 (Additional file 3). The ABBA-BABA tests indicated admixture between *S. tumulosa* and *S. pavida* lineages (α = 3.0, Z = 3.44–4.10, D = 0.11; tests 10, 18). Eleven of thirteen individuals of *S. tumulosa* (both clades a and b) appeared to be strongly admixed with *S. pavida* (α = 3.0, Z = 3.11–4.55, D = 0.11–0.18; tests 11, 14–17, 19–24, Additional file 3). Upon further examination with partitioned D-statistics, introgression appeared to have occurred from *S. pavida* into both *S. tumulosa* clades (α = 3.0, Z = 2.8–3.0, D = -0.14–0.15, Additional file 3).

### Clade 5C

DAPC and Structure analyses supported eight species in *Sinularia* clade 5C (*S. acuta/abrupta*, *S. densa, S. exilis, S. lochmodes, S. maxima, S. penghuensis/daii, S. slieringsi,* and *S. wanannensis*); *S. abrupta* was not distinguished from *S. acuta*, and the holotype of *S. daii* was placed with *S. penghuensis*. In addition, five individuals identified as *S. penghuensis*, including the holotype and paratypes, grouped with *S. slieringsi*. The optimal number of *K* clusters suggested by the DAPC analyses was eight (BIC = 377, Additional file 1: Figure S3), and the DAPC plot revealed no overlap among these distinct clusters (Fig. 5b). Further support for group assignment can be seen in the assignment plots, as all individuals were successfully re-assigned into their respective clusters (Additional file 1: Figure S5). In addition, the Distruct plot clearly illustrated little to no admixture between these eight species (Fig. 3), except that both individuals of *S. exilis* appeared to be admixed with at least three different species, including *S. densa, S. maxima,* and *S. wanannensis.* Individuals of *S. abrupta* (D329) and the holotype of *S. wanannensis* (Z34695) whose *28S* barcode sequences were incongruent with their *mtMutS* haplotypes (Additional file 1: Figure S1) also showed some evidence of admixture with *S. maxima* and *S. densa*, respectively.

It is possible that *S. slieringsi* represents two cryptic species, although results are not conclusive. Upon further Structure analysis, little to no admixture was revealed between two sub-clades of *S. slieringsi*; however, two individuals (one of them a paratype of *S. penghuensis*, Z34681) appeared admixed (Fig. 3). Following a one-species model (MLE = -1298), DAPC+ 1 was the second most likely (MLE = -1543) species model according to BFD* analyses (Table 3). The DAPC+ 1 model included species designated by DAPC, plus the two groups of *S. slieringsi*. Second, individuals of *S. slieringsi* formed two separate groupings in the DAPC plot, although there was some overlap among individuals (Fig. 5b). *S. slieringsi* also split into two reciprocally monophyletic clades in the species tree phylogeny (Fig. 4b), but not in the concatenated RAxML or *mtMutS + 28S* phylogenies (Fig. 3; Additional file 1: Figure S2).

**Fig. 7:**
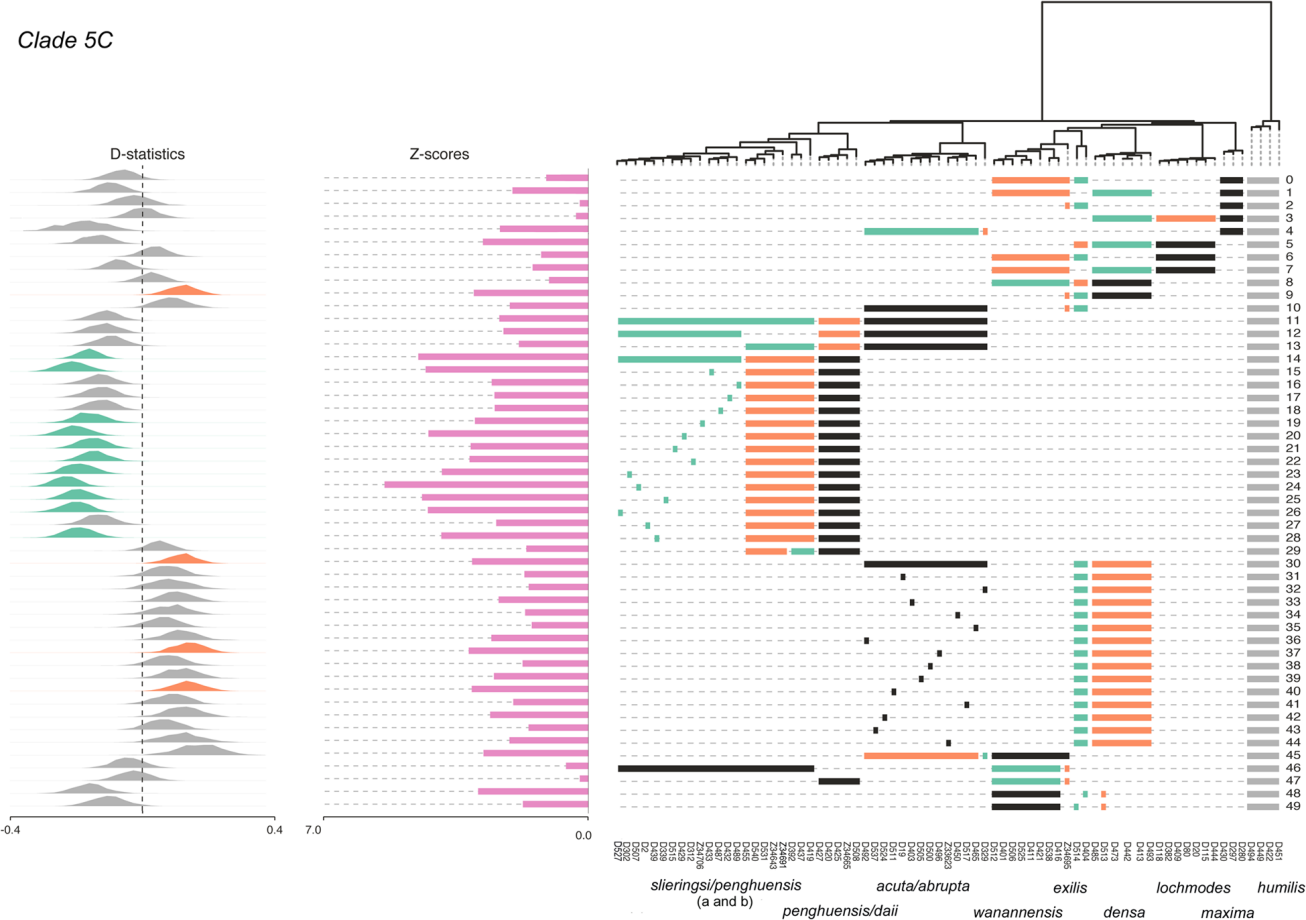
D-statistic tests for admixture in Dongsha Atoll *Sinularia* clade 5C. Test numbers are listed on the right for each 4-taxon test (((p1, p2), p3), p4). Horizontal bars below the tips of the tree indicate which taxa were included in each test. *S. humilis* was set as the outgroup for all tests (indicated by gray bars). Tests are configured to ask whether P3 (black bars) shares more derived SNPs with lineage P1 (green bars) relative to P2 (orange bars). As illustrated to the left, Z scores are bar plots and D-statistics are histograms. Histograms are green for significant gene flow between P1 and P3 (BABA) and orange for significant gene flow between P2 and P3 (ABBA). D-statistics that were not significant are gray. Significance was assessed at an alpha level of 3.0 (i.e., when D deviates > 3.0 standard deviations from zero)

Fifty separate ABBA-BABA tests were run on clade 5C (Fig. 7, Additional file 2). The average number of loci shared across taxa in each test was 1745 ± 216 (Additional file 3). The ABBA-BABA tests indicated admixture between the *S. penghuensis/S. daii clade* and one clade, clade b, of *S. slieringsi* (α = 3.0, Z scores =4.51, D = -0.15; test 14). Most individuals in the latter clade (which included the holotype of *S. penghuensis*) appeared to be strongly admixed as evidenced by ABBA-BABA tests (α = 3.0, Z = 3.1–5.5, D = -0.13– -0.20; tests 15, 19–26, 28). As suggested by the Structure analysis, the holotype of *S. wanannensis* (Z34695) showed strong admixture with *S. densa* (α = 3.0, Z = 3.13, D = 0.14; test 9), but not with *S. maxima* (α = 3.0, Z = 0.25, D = -0.01; test 2). It was also not admixed with either *S. penghuensis* or *S. slieringsi* (α = 3.0, Z = 0.24–0.61, D = -0.03 –0.01; tests 46–47), even though it shared the same *mtMutS* haplotype as both of those species. Although *S. abrupta* D329 showed some evidence of admixture with *S. maxima* and *S. densa* in the Structure analysis, the ABBA-BABA tests indicated that it was not a hybrid (α = 3.0, Z = 1.59–2.78, D = 0.10–0.18; tests 32, 45). The *S. abrupta* specimen, Z33623, that shared a *28S* sequence with *S. densa* was also not significantly admixed with that species (α = 3.0, Z = 2.11, D = 0.13, test 44), whereas *S. abrupta* D19 was (α = 3.0, Z = 312, D = 0.15; test 40). One individual of *S. acuta,* D450, was also admixed with *S. densa* (α = 3.0, Z = 3.21, D = 0.16; test 37). Notably, the Distruct plot showed strong admixture of the two *S. exilis* specimens with *S. densa, S. maxima,* and *S. wanannensis* (Fig. 3)*.* However, ABBA-BABA tests suggested that these individuals were not significantly introgressed with those or any other species (α = 3.0, Z = 0.25–2.96, D = -0.15–0.04; tests 0–2, 5–6, 8, 48–49). Overall, the ABBA-BABA tests indicated that at least 15 individuals were significantly introgressed with other species at an α = 3.0; however, we note that D statistics for several other tests also deviated considerably from 0, although they were not significant at α = 3.0.

## Discussion

### Species delimitations

A few different species delimitation methods based on RADseq data resulted in incongruence in the number of species suggested to be present among those sampled at Dongsha Atoll in each of *Sinularia* clades 4 and 5C. BFD* analysis indicated that for each clade the most likely model was a one-species model, whereas DAPC indicated that four species were present in clade 4 and eight were present in clade 5C. It seems unlikely that only a single species exists in each of clades 4 and clade 5C because there is little overlap among groups in the DAPC plots and there is strong genetic structure shown in the Structure analyses. Furthermore, there are well-supported clades in both ML and species trees, many of them congruent with distinct morphologies [8]. The suggestion by BFD* analysis of only one species in each of the two clades is likely spurious due to the relatively few loci included in those analyses. Because of the computational time it took to run each SNAPP species-delimitation model, only complete datasets were used, i.e., those that included no missing data, and these contained relatively few SNPs (< 200).

All other species delimitation analyses supported four or five species of *Sinularia* in clade 4 and eight or nine in clade 5C among the samples that were sequenced. Within clade 4, RADseq and both barcoding markers discriminated *S. humilis*, *S. ceramensis* and *S. pavida* from one another and from all specimens identified as *S. tumulosa*. Both the *mtMutS + 28S* barcode and RADseq results further delineated two distinct clades within *S. tumulosa*, with hybridization tests suggesting that both are admixed with *S. pavida*. Within clade 5C, species delimitation analyses clearly distinguished *S. lochmodes*, *S. densa*, *S. wanannensis* and *S. maxima* from all other species, and each of those morphospecies also had unique haplotypes at both barcoding loci. *S. acuta* was similarly delineated from all other morphospecies with the notable exception of *S. abrupta*. Two of three individuals of *S. abrupta* shared a *mtMutS* haplotype with *S. acuta*, and could not be distinguished from that species in the DAPC and Structure analyses. A third colony identified as *S. abrupta* (D329) showed signs of admixture in both Structure and ABBA-BABA analyses, suggesting a possible hybrid origin. As *S. acuta* and *S. abrupta* differ markedly in both colony growth form and sclerite morphology [8], the apparent lack of genetic distinction between these two morphospecies warrants further study.

The remaining morphospecies in clade 5C could not be separated clearly using either barcode marker, and the multilocus analyses suggested that admixture might contribute to the difficulty distinguishing them. Although both individuals of *S. exilis* had unique *mtMutS* haplotypes, they shared *28S* genotypes with *S. densa* and S*. wanannensis*. Phylogenetic analyses placed *S. exilis* as the sister to *S. wanannensis*, but Structure suggested considerable admixture with both *S. maxima* and *S. densa*. As ABBA-BABA tests did not strongly support a hybrid origin of *S. exilis*, incomplete lineage sorting may better explain why this species shares genotypes with other species in the clade. Hybridization was, however, supported as a possible explanation for the confusing relationship between *S. penghuensis* and *S. slieringsi*, two morphospecies that shared several different *mtMutS* haplotypes, one of which was also shared by *S. daii*. Five colonies of *S. penghuensis*, including the holotype (ZMTAU Co 34706) and two paratypes (ZMTAU Co 34643, Co 34681), shared a *28S* genotype with *S. slieringsi,* while the other four shared a very different *28S* genotype with the holotype of *S. daii* (ZMTAU Co 34665). Multilocus species delimitation analyses separated the latter four *S. penghuensis* plus *S. daii* from a large clade that included all *S. slieringsi* plus the *S. penghuensis* type specimens. Structure and DAPC further separated that large clade into two sub-clades, suggestive of possible cryptic species. ABBA-BABA tests indicated that a majority of the individuals of *S. slieringsi* and *S. penghuensis* in one of those two sub-clades are admixed with the *S. penghuensis-S. daii* clade.

### Evidence for hybridization in Sinularia

It is important to use a phylogenetic framework in assessments of introgressive hybridization. A species that appears admixed could have a close relative harboring a stronger signal of admixture [26], and if that species is not included in analyses, then the admixture will be incorrectly attributed to a closely-related taxon [23, 25, 90]. In addition, it can be challenging to distinguish introgression between two species from “secondary genomic admixture”, which occurs when one species shares recent ancestry with a true hybridizing lineage, thus causing that species to also appear as if it were admixed [25, 26]. Although the current study sequenced most of the clade 4 and 5C morphospecies that occur at Dongsha Atoll, there were at least two morphospecies in each clade that were not included in the analyses. In addition, there are other *Sinularia* species in phylogenetically distinct clades that also inhabit this atoll, and those too were not included in the analyses. While our results provide evidence for hybridization, we acknowledge the possibility of incorrectly attributing admixture to a close relative of the true hybridizing lineage, as not all possible *Sinularia* morphospecies were included in these analyses.

Because of the difficulties interpreting ABBA-BABA tests even when using a phylogenetic framework [26], it is best to focus on the strongest signals of admixture [28]. The signal of introgression was strong in clade 4, with both clades of *S. tumulosa* showing admixture with *S. pavida*; no other hybridization tests in this clade were significant, with D-statistics centered around zero and Z scores fairly low. In contrast, it was more difficult to confidently determine which *Sinularia* species are hybridizing with others in clade 5C, which could be due to the use of an incomplete phylogeny. There were some cases where the signal of admixture was strong (e.g., *S. acuta* with *S. densa, S. slieringsi* with *S. penghuensis)* and supported by other tests in addition to ABBA-BABA. However, there were also cases where species appeared admixed, but the ABBA-BABA results were not significant at an alpha of 3.0. For example, Structure analyses suggested there was considerable admixture between *S. wanannensis* and *S. exilis,* and at least one *S. exilis* individual had the same *28S* barcode as *S. wanannensis*, but the ABBA-BABA tests were not significant*.* Perhaps with more *S. exilis* individuals in the analyses, or with the addition of the missing morphospecies of *Sinularia*, a more complete picture would emerge as to whether these species share genes as a result of incomplete lineage sorting or hybridization. Because the current phylogenetic analysis did not include all species, it is possible that the species identified as the source of introgressed alleles may simply be close relatives of the actual parental species. Nevertheless, the phylogenetic framework was a useful approach in determining that hybridization appears to be an important process in the diversification of this speciose group of soft corals.

Incongruence between the RAxML and SNAPP species trees (both built using 25% missing data) may provide further support for hybridization among *Sinularia* species. In the clade 4 SNAPP species tree, *S. ceramensis* was sister to a clade of *S. pavida* plus *S. tumulosa,* whereas in the ML tree built using concatenated RAD loci, *S. ceramensis* was sister to *S. pavida.* In clade 5C, *S. acuta* was sister to *S. penghuensis* and *S. slieringsi* in the ML tree, but sister to all other species in the SNAPP species tree. Notably, relationships that differed between analyses showed evidence of admixture in the ABBA-BABA results. Although incomplete lineage sorting can lead to discordance between phylogenies built using concatenated data vs. species tree methods [29, 61, 66], hybridization has also been shown to produce incongruence among gene trees [29, 62]. Introgressive hybridization may also explain the alternative topologies recovered in the SNAPP species tree. Johnston et al. [53] suggested that alternative trees emerging from SNAPP analyses of corals in the genus *Porites* could be due to introgressive hybridization, incomplete lineage sorting, or contamination of loci by symbionts (e.g., Symbiodiniaceae). The species that displayed different relationships in the SNAPP trees were shown to be potentially hybridizing by our ABBA-BABA tests, suggesting that alternative SNAPP topologies may be driven by the presence of introgressive hybridization. Results further lend support to the idea that diversification of species-rich lineages may not be a solely bifurcating process. As such, phylogenetic tree reconstructions that include taxa that do not follow the usual assumption of a bifurcating process of evolution can lead to incongruence among gene trees and contribute to difficulties in resolving phylogenies.

### Morphospecies versus genetic data

Incongruence between morphological and molecular evidence for species boundaries is common in corals (e.g., [36, 37, 58]). Contributing factors include environmental plasticity (e.g., [80]) and frequent homoplasy of morphological characters (e.g., [36]), as well as the slow rate of mitochondrial gene evolution that has made “universal” molecular barcodes such as COI relatively invariant among congeneric species [50, 71, 96]. When barcodes fail to discriminate distinct morphospecies it may be because the markers lack appropriate variation, or, alternatively, because morphological variation within a species has been incorrectly interpreted as evidence of a species boundary [73]. Attempts to integrate the two different sources of evidence have met with some success, as demonstrated by Benayahu et al. [8]. By combining assessment of morphology with a character-based barcoding approach, that study identified at least 27 species of *Sinularia* from Dongsha Atoll*,* including those used in the current study. In several cases in which distinct morphotypes shared identical *mtMutS* haplotypes, however, the lack of congruence was attributed to invariance of the barcode marker (i.e., incomplete lineage sorting), and thus species were delimited using morphological characters. In two such cases, species delimitation methods using RADseq data also failed to support the genetic distinction between discrete morphotypes, namely *S. acuta* and *S. abrupta,* and *S. slieringsi* and *S. penghuensis.* Moreover, our inclusion of type specimens of *S. penghuensis* and *S. daii* as taxonomic references revealed no genetic distinction between the material identified here as *S. slieringsi* and the *S. penghuensis* types, or between the holotype of *S. daii* and additional colonies identified as *S. penghuensis*. In addition, a colony from Palau identified as *S. verruca* (R41341) could not be distinguished genetically from one of the two clades of *S. tumulosa*. Clearly, additional taxonomic work integrating both morphological and molecular approaches will be necessary to clarify the relationships among these taxa.

Morphological discrimination of species is complicated in *Sinularia* and many other soft corals due to the continuous nature of many of the characters used to diagnose species. Colony growth forms and the intricate shapes of sclerites are difficult to quantify and may present a continuum of variation, as do morphometric characters such as the sizes of sclerites commonly reported in the literature (e.g. [111]). Many of the species examined here, including *S. tumulosa*, *S. verruca, S. acuta* and *S. daii*, were described from single exemplars [6, 67, 76], and therefore no data exist on the possible range or limits of morphological variation they exhibit, which potentially confounded efforts to discriminate them from other similar species. Hybridization also offers a possible explanation for the lack of congruence between morphological and molecular determinations of species identity. As has been suggested for some coral genera, hybridization can lead not only to morphologically distinct or intermediate phenotypes (*Porites*: [38]; *Acropora*: [107, 109, 112]), but also to F1 hybrids that exhibit characters of both parental species [101, 112]. In a naturally occurring hybrid zone in Guam, for example, F1 hybrids of *S. maxima* and *S. polydactyla* were found to contain a mix of sclerites resembling those of both parental species [101]. Perhaps mechanisms such as these add to the confusion in morphospecies identification, which is often pervasive in *Sinularia* and other octocorals. Although it is currently unknown whether or not hybridization generates new species or asexual lines in the genus *Sinularia,* admixture between *Sinularia* species occurs, and perhaps contributes to the range of morphotypes observed in the genus.

### Utility of DNA barcoding in corals

As numerous other studies have now cautioned, none of the single-gene molecular barcodes currently used to help guide species identifications in octocorals successfully delimit all species [3, 48, 71, 72, 79], particularly when genetic distance thresholds are used to decide species boundaries. For example, in *Sinularia* clade 5C only four MOTUs were identified among eight morphospecies using the *28S* marker, whereas *mtMutS* resolved eight MOTUs, but not all of them were congruent with morphospecies and RADSeq delimitations. Such lack of concordance among different molecular markers is not uncommon in corals and in recently diverged clades more generally [72, 81, 85, 108]. For octocorals, a consensus has emerged that mitochondrial and *rDNA* barcodes may be useful in species assessments for some taxa (e.g., [72, 79]), but not all. Multiple markers as well as other lines of evidence need to be considered when delimiting species [73]. Genomic approaches such as RADseq are effective [48, 79], but still prohibitively expensive and impractical to use for the routine species identification work required of biodiversity surveys. Alternatively, once species boundaries have been validated using such approaches, it may be possible to identify morphological or simple molecular characters that are species-diagnostic. As discussed above, however, the continuum of variation in morphological traits of corals complicates the search for diagnostic characters, and in some recently discriminated octocoral taxa none have yet been identified [73].

Single-gene barcode markers such as *mtMutS* and *28S* offer diagnostic nucleotide characters that can be used to identify cryptic taxa [71]. When Benayahu et al. [8] applied a character-based *mtMutS* barcode to the *Sinularia* species found at Dongsha, the only morphospecies that could not be discriminated were the same ones for which the current study also found incongruence between RAD clades and morphospecies designations: *S. acuta*, *S. abrupta*, *S. penghuensis, S. daii* and *S. slieringsi*. A compound, character-based barcode that combines *mtMutS* with *28S*, however, yields diagnostic characters that discriminate each of the *Sinularia* clades identified by RADseq, including both sub-clades of *S. tumulosa* and *S. slieringsi* (Additional file 1: Figure S6). Once species boundaries have been validated using integrated, genomic approaches, such as those applied here, use of simple character-based barcodes to identify morphologically cryptic species may be more time- and cost-effective than genomic approaches. Assignment of character-based barcodes, however, requires a priori recognition of species boundaries, as well as screening of a sufficient number of individuals to identify polymorphic characters.

### Future research directions

Further investigation is needed to determine the evolutionary processes responsible for generating the high species diversity in the genus *Sinularia,* but a necessary first step is to understand how many species there are and where they are distributed. With accurate species identifications utilizing both classical taxonomy and advanced genomic techniques, it will be possible to address questions pertaining to how and when *Sinularia* diversified into coral reef environments and why species in this genus appear to be so successful at co-existing. One intriguing question is whether the high diversity of *Sinularia* was generated in sympatry through mechanisms such as hybrid speciation or whether species have diverged in allopatry and then colonized the same reefs. With the advent of new genomic techniques such as RADSeq and target-capture genomics (e.g., [83]), we can begin to examine how pervasive hybridization is on coral reefs, particularly because F1 hybrids and their progeny may be more fit than the parent populations, and hybrid vigor may help in the maintenance and resilience of coral reef diversity [101]. Using a genomic approach can help to guide species delimitation while simultaneously shedding light on the processes generating diversity in this genus— just one of many hyperdiverse coral lineages (e.g., the scleractinians *Acropora, Porites, Pocillopora* and the octocorals *Dendronephthya, Lobophytum* and *Sarcophyton*) in which ecologically similar congeners co-occur in high numbers on coral reefs throughout the Indo-Pacific.

## Conclusions

The results presented here suggest that the answer to both of the questions that originally motivated this study is ‘yes’. In most—but not all—cases, multilocus species delimitation analyses validated the species boundaries between morphologically distinct taxa that nonetheless shared identical *mtMutS* or *28S* barcode sequences. In some cases, barcodes appeared to be shared among morphospecies as a result of incomplete lineage sorting at mitochondrial or rDNA loci. In other cases, including ones in which multilocus analyses did not support the genetic distinction of individuals that differed morphologically, there was evidence for admixture and introgressive hybridization among species. Finally, in several cases in which morphospecies could not be distinguished genetically but there was no evidence for admixture or hybridization, it will be necessary to validate whether or not the observed morphological differences are indicative of species boundaries or intraspecific variation.

Delimiting species is a critical first step in documenting the biodiversity of ecologically important reef inhabitants, such as species in the genus *Sinularia*. This study demonstrates the utility of using genomic approaches to delimit species within a hyperdiverse lineage of soft corals and to examine simultaneously whether hybridization may be contributing to its diversification. Although there was some incongruence among datasets and species delimitation methods, we can confidently conclude that the sequenced individuals represent at least four species of *Sinularia* in clade 4 and eight species in clade 5C [70], with the potential for one additional cryptic species in each clade. The results point to hybridization as an evolutionary pathway to diversification in *Sinularia*, and suggest that this mechanism may produce hybrids with morphologies intermediate to those of their parental species, contributing to the difficulty of assigning species based on morphology in this and other coral genera [38, 40, 74, 101]. Furthermore, our results raise the possibility that hybrid speciation (i.e., reticulate evolution via introgressive hybridization) is one mechanism that has contributed to the diversity of octocorals.

## Methods

### Specimen collection and preparation

Colonies of *Sinularia* were collected using SCUBA during biodiversity surveys conducted at Dongsha Atoll Marine National Park (Taiwan) in 2011, 2013 and 2015 [8]. Collections were made at 13 sites in the shallow fore-reef zone (3–21 m) surrounding the 25-km diameter atoll (see Fig. 1 in [8]). During the 2015 survey we specifically targeted common morphospecies belonging to clades 4 and 5C [70]. Wedges of tissue (usually < 50 cm^2^ in area) were cut from the colony perimeter and pried off the substrate; colonies typically heal and regenerate this lost tissue within a few months (pers. obs.). Following collection, small subsamples of tissue (~ 100 mg) were preserved in 95% EtOH, and the remainder of the specimen was preserved in 70% EtOH. All vouchers have been deposited in the Steinhardt Museum of Natural History, Tel Aviv University, Israel (ZMTAU, Additional file 4). To identify morphospecies, sclerites were isolated from colonies by dissolving tissue in 10% sodium hypochlorite and examined using either light microscopy or, when necessary, scanning electron microscopy (SEM) [8]. The morphological IDs were made by direct comparison to type material when available. Specimens belonging to 13 *Sinularia* morphospecies, four from clade 4 and nine from clade 5C, were selected for further species delimitation analyses. Seven specimens collected in previous biodiversity surveys of the Penghu Archipelago, Taiwan [7] and Palau [72], including original type material of *S. penghuensis* (ZMTAU Co 34643 (=Z34643), Co 34706 (=Z34706), Co 34681 (=Z34681)), *S. wanannensis* (ZMTAU Co 34695 (=Z34695)) and *S. daii* (ZMTAU Co 34665 (=Z34665)), were also included as taxonomic references (Table 1; Additional file 4).

DNA was extracted from 95 *Sinularia* specimens, and quantified using a Qubit v 2.0 fluorometer (Broad Range Assay Kit). Quality was assessed by running 100 ng of DNA for each sample on a 1% agarose gel, and checked with a NanoDrop spectrophotometer. Concentration of high-quality (230/260 and 260/280 ratios > 1.8) DNA was normalized to 20 ng per ul and sent to Floragenex Inc. (Eugene, OR) for RADSeq library preparation. DNA libraries were constructed for each of the 95 samples using the 6-cutter *PstI* enzyme, and then split into two for sequencing 100 bp SE reads on two full lanes of an Illumina HiSeq2500 (University of Oregon’s Genomics and Cell Characterization Core Facility lab). In addition to RADseq, two gene regions (*mtMutS*, *28S* rDNA) used widely for barcoding in octocorals were PCR-amplified and Sanger-sequenced using published primers and protocols [71, 72].

### Phylogenetic inference and species delimitation using DNA barcodes

*mtMutS* and 28S sequences of the *Sinularia* species were each aligned using the L-INS-i method in MAFFT [56], and pairwise genetic distances (Kimura 2-parameter) among sequences were calculated using the DNADist program in PHYLIP v. 3.69 [35]. MOTHUR v 1.29 [94] was used to delimit molecular operational taxonomic units (MOTUs) based on a genetic distance threshold of 0.3% applied to *mtMutS* and 28S separately as well as combined in a concatenated dataset (e.g., [72]). Phylogenetic trees were constructed separately for *mtMutS* and *28S* rDNA and for the concatenated dataset using maximum likelihood methods (Garli; [117]) (Additional file 1: Figures S1 and S2). jModelTest [19] was used to identify the best models of evolution (AIC criterion) to use in these analyses (*mtMutS*: HKY + G; *28S rDNA*: HKY + I).

### Phylogenetic inference and species delimitation using RADSeq data

We produced several different RADSeq locus datasets using pyRAD v 3.0 [24] with different parameter combinations, following recommendations in the pyRAD and ipyRAD tutorials [24, 27] and previous studies [48, 79]. Datasets chosen for species delimitation and phylogenetic analyses included those loci from pyRAD parameter settings that maximized the number of phylogenetically informative sites, while minimizing missing data and eliminating the potential for paralogous loci (Table 2, see Additional file 5 for more details). Thus, for most analyses, we used data produced from the following parameter settings: *c* 0.85, *p* 0.25, and *m* 0.75 for each of clades 4 and 5C.

RAxML v8 [102] was used to create maximum likelihood (ML) phylogenies for clades 4 and 5C. A GTR + G + I model as suggested by the Akaike Information Criterion [(AIC = 371918), JModelTest v2, [19]] was used. A total of 20 ML searches and 200 bootstrap replicates were performed using rapid bootstrapping on concatenated loci. RAxML analyses were performed (12 analyses per clade) for each of the different datasets produced by pyRAD with different parameter combinations and clustering thresholds (Table 2, Additional files 5 and 6).

Discriminant Analysis of Principal Components (DAPC) was performed using the package ‘adegenet’ v2.0 in R [54, 84] to explore genetic structure in both clades 4 and 5C. The DAPC method, used previously in species delimitation analyses [79], forms clusters based on genetic similarity of each multilocus genotype, without considering a model of evolution. We first used the function *find.clusters* to find the best number of *K* genetic clusters in unlinked SNP datasets for each clade. *Find.clusters* was performed on *K =* 20 for each clade. The lowest value of the Bayesian Information Criterion (BIC) statistic was used to detect the optimal number of *K* clusters. These clusters were then analyzed using DAPC, which first transforms the data using principal components analysis and then performs a Discriminant Analysis on the retained principal components. The *optim.a.score* function was used to determine how many PC axes needed to be retained (Additional file 1: Figure S7). Six PCAs and four discriminant functions were retained for clade 4; and six PCAs and six discriminant functions were retained for clade 5C (Additional file 1: Figure S8). Scatterplots of discriminant functions were then created. We also used the function *assignplot* to visualize individual membership in each *K* cluster, which can help show the accuracy of the cluster assignments and identify any individuals that have high probabilities of membership in > 1 cluster.

The Bayesian model-based clustering approach, Structure v2.3 [82], was also used to infer the number of *Sinularia* species. The program clusters individuals based on genetic variation alone, without any other prior information such as geographic origin or population assignment. Structure was performed on unlinked SNP datasets for both *Sinularia* clades 4 and 5C, and run in parallel using StrAutoParallel v 1.0 [16] using an admixture model with correlated allele frequencies. Burnin was set to 250,000 followed by 1,000,000 MCMC generations. The inferred number of populations (*K*) was set from 1 to 8 for clade 4 and 1 to 10 for clade 5C; 20 runs of each *K* were conducted. Multiple runs of each *K* were aligned with CLUMPP v 1.2 [51], and the resulting *indivq* file was input into Distruct v. 1.1 [91] for graphical display of individual population assignments. The commonly used Δ*K* method [30] was not used in our study to identify an optimal *K* value because this method is known to be most successful at finding only the uppermost levels of genetic structure in a hierarchical system [30]. Initial tests of the Δ*K* method revealed *K* = 2 as the best model for each clade; however, analysis at *K* > 2 indicated strong genetic structure in both clades 4 and 5C. Therefore, we plotted the population structure assignments of *K = 4* for clade 4 and *K = 8* for clade 5C, because these were the number of genetic clusters suggested by DAPC analyses, and Structure analyses indicated little to no admixture between putative species at these *K* clusters. Then, following Gowen et al. [42], we analyzed successively smaller groups of potential species in separate analyses at *K* = 2. We plotted the results of *K* = 2 runs for the putative species, *S. tumulosa* and *S. slieringsi,* because Bayes Factor Delimitation with genome data (BFD*) analyses (see below), suggested the presence of additional species within each of *S. tumulosa* and *S. slieringsi.*

Coalescent-based SNAPP v 1.3 [13] analyses were used to test alternative species models for both clades 4 and 5C. Samples were assigned to the following alternative species models (Additional file 1: Figures S9 and S10): 1) one species (ONESP), 2) two species (TWOSPP), 3) DAPC clusters (DAPC), 4) DAPC clusters plus division of another clade (DAPC+ 1), 5) MOTU assignments based on *mtMutS* (MUTS), and 6) MOTU assignments based on *28S* rDNA (28S). In addition, three (THREESPP) and four (FOURSPP) species models were also tested for clades 4 and 5C, respectively. SNAPP analyses were performed in BEAST v 2.4.5 [11] with a path sampling of 48 steps (MCMC length = 100,000, pre-burnin = 1000) following Leaché et al. [64] and Herrera & Shank [48]. The *c*0.85, *m*1.0, *p*0.25 bi-allelic SNP datasets (175 SNPs for clade 4, 140 SNPs for clade 5C, no missing data) were used because of the long computational time it took for each SNAPP run. Marginal likelihood estimates were obtained for each different model run in SNAPP analyses. The different species delimitation models were then ranked using BFD* methods. Bayes Factors were calculated between each of two alternative models by subtracting the marginal likelihood estimates between two models, and then multiplying the difference by two (following [57, 64]).

SNAPP was also used to infer the species tree for each *Sinularia* clade. Three independent runs were performed on SNP data (MCMC length = 1,000,000, pre-burnin = 1000, samplefreq = 1000) using BEAST with default parameters for mutation rate, coalescent rate, and ancestral population sizes (following [48]). The *c*0.85, *m*0.75, *p*0.25 bi-allelic SNP datasets were used for species tree analyses. Acceptance probabilities were checked to ensure that tuning parameters were appropriate and the chain mixed well [22]. Log files were combined using Log Combiner v 1.1 and input into Tracer v1.6 [86]. Convergence and ESS > 200 were assessed using Tracer after a 10% burnin. Maximum clade credibility trees were generated with Tree Annotator v 2.3 [11]. Both the consensus tree and all tree topologies were drawn in DensiTree v2.2 [10].

### Hybridization tests

We calculated Patterson’s D statistics in ipyRAD v. 0.7.28 [27] to test for hybridization between species. Briefly, these tests calculate the proportion of ABBA and BABA site patterns, and excess of either is indicative of admixture rather than incomplete lineage sorting [23, 43]. Multiple 4-taxon tests were generated for both clades 4 and 5C (Additional file 2). For both clades, *S. humilis* was set as the outgroup (‘p4’). For tests that included multiple individuals per lineage, SNP frequencies were pooled. For tests performed on clade 4, each species was set as ‘p3’ and all possible 4-species combinations were tested. For clade 5C, all possible 4-species combinations were tested in each of two sub-clades (i.e., the *S*. *slieringsi-S. penghuensis*-*S. acuta* clade and the *S*. *wanannensis- S. exilis-S. lochmodes-S. densa*-*S. maxima* clade). Additional tests were conducted either when Structure results indicated potential admixture or there was incongruence between the different molecular markers (i.e., 28S, *mtMutS*, RAD). When test results were significant at the species level, further tests were performed to determine if particular individuals within the lineage were admixed. Significance of each test was determined by performing 1000 bootstrap replicates in which loci were resampled with replacement. Both D statistics and Z statistics, which represent the number of bootstrap standard deviations (alpha = 3.0) that D statistics deviate from zero [34], are reported. Following D-statistic tests, partitioned D-statistic tests were performed for a few cases to examine the direction of introgression. Tests were conducted and figures were plotted following the ipyRAD ABBA-BABA cookbook in Jupyter Notebook [60].

## Additional files

Additional file 1: **Figure S1**. ML trees of barcode data. ML phylogenies generated using mtMutS and 28S rDNA barcode data. **Figure S2.** ML tree of combined barcode data. ML phylogeny generated using a mtMutS + 28S rDNA dataset. **Figure S3.** BIC plots. Bayesian Information Criteria indicating optimal number of K clusters. **Figure S4.** Probability of membership plot for Clade 4. Individual reassignments based on discriminant functions. **Figure S5.** Probability of membership plot for Clade 5C. Individual reassignments based on discriminant functions. **Figure S6.** Character based barcodes. Species-diagnostic nucleotide characters.** Figure S7.** Optimal a-scores. A-scores indicate the optimal number of principal components to retain. **Figure S8.** Discriminant analysis eigenvalues. Total number of discriminant eigenvalues retained. **Figure S9.** Clade 4 species models and RAxML phylogeny. Species models used in BFD* analysis with phylogeny constructed using RAxML on RADSeq data. **Figure S10.** Clade 5C species models and RAxML phylogeny. Species models used in BFD* analysis with phylogeny constructed using RAxML on RADSeq data. (ZIP 22000 kb)

Additional file 2: ABBA-BABA tests. List of ABBA-BABA tests performed in ipyRAD. (TXT 13 kb)

Additional file 3: ABBA-BABA test results. Results of ABBA-BABA tests for both clades 4 and 5C. (XLSX 65 kb)

Additional file 4: *Sinularia* specimen data. Table of specimen data with morphospecies identifications and assigned species delimitations based on MOTUs and RADSeq data. (XLSX 19 kb)

Additional file 5: Supplemental Methods. Description of additional methods [15, 63, 65, 95, 104, 110]. (PDF 79 kb)

Additional file 6: Concatenated nexus files of all phylogenies constructed using RAxML on different pyRAD RADSeq datasets. (TXT 141 kb)

## Abbreviations

AFLP: Amplified fragment length polymorphism
BEAST: Bayesian evolutionary analysis by sampling trees
BFD*: Bayes factor delimitation (* with genomic data)
DAPC: Discriminant analysis of principal components
DNA: Deoxyribonucleic acid
FOURSPP: Four species
MCMC: Markov chain Monte Carlo
MLE: Marginal likelihood estimate
MOTU: Molecular operational taxonomic unit
mtMutS: Mitochondrial mutS-like protein
ONESP: One species
PC: Principal components
PCA: Principal components analysis
RADSeq: Restriction site-associated sequencing
RAxML: Randomized axelerated maximum likelihood
rDNA: Ribosomal deoxyribonucleic acid
SNAPP: SNP and AFLP package for phylogenetic analysis
SNP: Single nucleotide polymorphism
THREESPP: Three species
TWOSPP: Two species
ZMTAU Co: Zoological Museum Tel Aviv University, Coelenterates
