# Supplementary material for: A next generation approach to species delimitation reveals the role of hybridization in a cryptic species complex of corals
Source: BMC Evol Biol. 2019 Jun 6;19:116. doi: 10.1186/s12862-019-1427-y (PMC6555025; doi:10.1186/s12862-019-1427-y)
Supplement: Supplementary file 1 — Figure S1. ML trees of barcode data. ML phylogenies generated using mtMutS and 28S rDNA barcode data. Figure S2. ML tree of combined barcode data. ML phylogeny generated using a mtMutS + 28S rDNA dataset. Figure S3. BIC plots. Bayesian Information Criteria indicating optimal number of K clusters. Figure S4. Probability of membership plot for Clade 4. Individual reassignments based on discriminant functions. Figure S5. Probability of membership plot for Clade 5C. Individual reassignments based on discriminant functions. Figure S6. Character based barcodes. Species-diagnostic nucleotide characters. Figure S7. Optimal a-scores. A-scores indicate the optimal number of principal components to retain. Figure S8. Discriminant analysis eigenvalues. Total number of discriminant eigenvalues retained. Figure S9. Clade 4 species models and RAxML phylogeny. Species models used in BFD* analysis with phylogeny constructed using RAxML on RADSeq data. Figure S10. Clade 5C species models and RAxML phylogeny. Species models used in BFD* analysis with phylogeny constructed using RAxML on RADSeq data. (ZIP 22000 kb) [file 12862_2019_1427_MOESM1_ESM.zip › 12862_2019_1427_MOESM3_ESM.pdf]

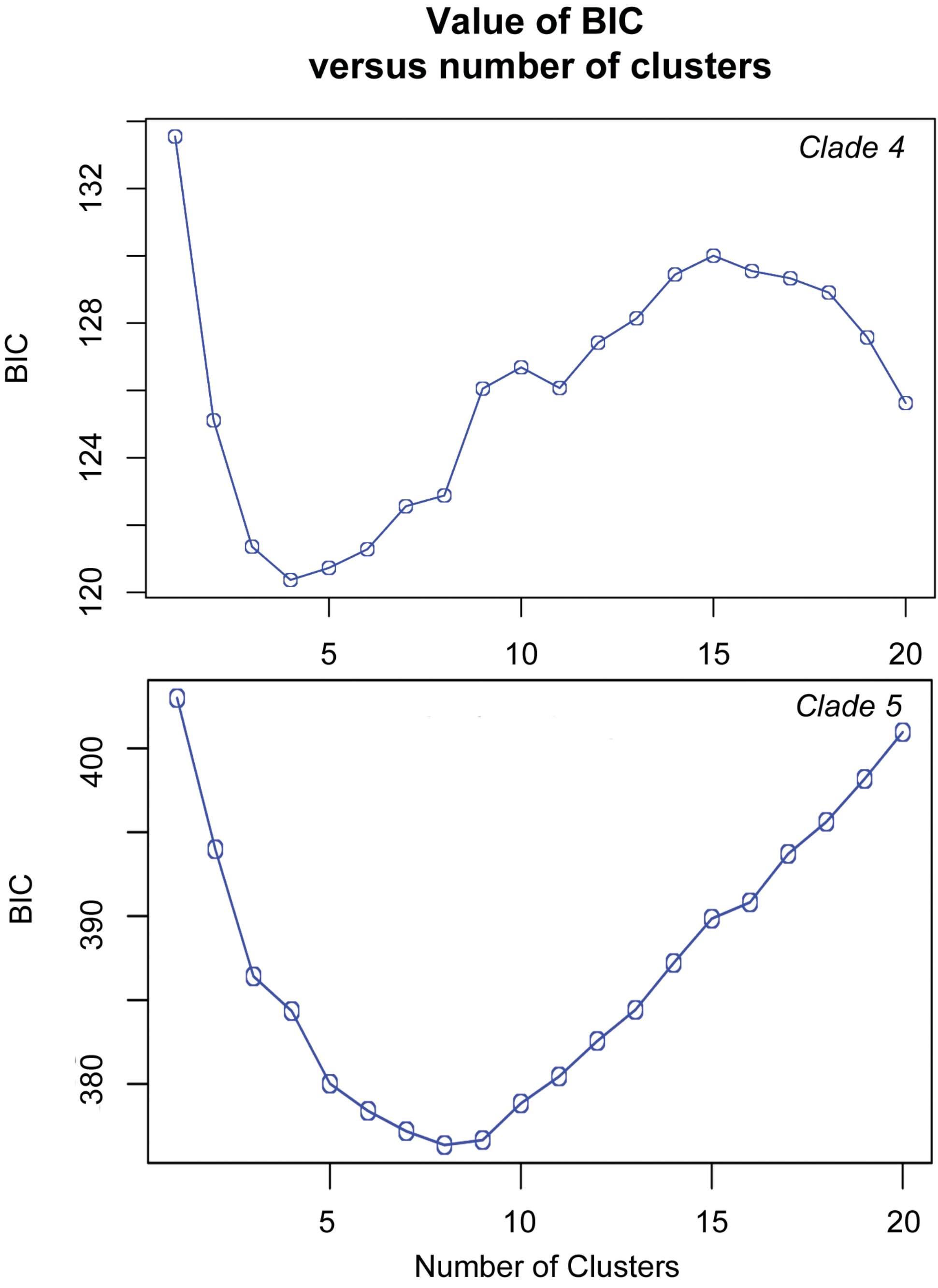

Bayesian Information Criterion (BIC) indicating the optimal number of k clusters.The lowest BIC often is determinant of the best number of k clusters.
