# Supplementary material for: A next generation approach to species delimitation reveals the role of hybridization in a cryptic species complex of corals
Source: BMC Evol Biol. 2019 Jun 6;19:116. doi: 10.1186/s12862-019-1427-y (PMC6555025; doi:10.1186/s12862-019-1427-y)
Supplement: Supplementary file 1 — Figure S1. ML trees of barcode data. ML phylogenies generated using mtMutS and 28S rDNA barcode data. Figure S2. ML tree of combined barcode data. ML phylogeny generated using a mtMutS + 28S rDNA dataset. Figure S3. BIC plots. Bayesian Information Criteria indicating optimal number of K clusters. Figure S4. Probability of membership plot for Clade 4. Individual reassignments based on discriminant functions. Figure S5. Probability of membership plot for Clade 5C. Individual reassignments based on discriminant functions. Figure S6. Character based barcodes. Species-diagnostic nucleotide characters. Figure S7. Optimal a-scores. A-scores indicate the optimal number of principal components to retain. Figure S8. Discriminant analysis eigenvalues. Total number of discriminant eigenvalues retained. Figure S9. Clade 4 species models and RAxML phylogeny. Species models used in BFD* analysis with phylogeny constructed using RAxML on RADSeq data. Figure S10. Clade 5C species models and RAxML phylogeny. Species models used in BFD* analysis with phylogeny constructed using RAxML on RADSeq data. (ZIP 22000 kb) [file 12862_2019_1427_MOESM1_ESM.zip › 12862_2019_1427_MOESM6_ESM.pdf]

mutS

Clade 4	M156	M168	M179	M200	M204	M217	M253	M264	M294	M307	M343	M365	M414	M429	M443	M504	M548	M573	M630	M631	M666	M716	M726
4.1 humilis	G	A	G	**C**	C	G	**A**	**C**	T	C	G	G	**C**	**C**	C	C	**A**	**T**	**T**	T	**A**	T	**T**
4.2 pavida	G	G	G	T	C	G	G	T	T	C	G	**A**	T	T	C	**T**	G	C	C	**C**	G	T	C
4.3 ceramensis	**A**	G	**R**	T	**T**	**A**	G	T	**C**	C	G	G	T	T	**M**	C	G	C	C	T	G	T	C
4.4a tumulosa a	G	R	G	T	C	**A**	G	T	T	C	**A**	G	T	T	C	C	G	C	C	T	G	T	C
4.4b tumulosa b	G	A	G	T	C	G	G	T	T	**Y**	G	G	T	T	C	C	G	C	**T**	T	G	**C**	C

28S

Clade 4	S073	S076	S106	S109	S122	S166	S171	S185	S206	S225	S232	S289	S315	S362	S363	S365	S366	S447	S454	S515	S516	S530	S531	S532	S533	S534	S535	S536	S537	S538	S539	S540	S541	S542	S543	S544	S545	S551
4.1 humilis	**C**	**G**	A	**G**	G	**T**	**T**	G	C	**C**	**G**	**G**	C	C	A	G	**C**	**A**	**C**	A	**T**	**G**	**C**	**G**	**C**	**T**	**T**	**C**	**G**	**T**	**C**	**T**	**C**	**G**	**A**	C	G	C
4.2 pavida	T	A	A	C	G	C	G	G	C	T	A	T	C	C	A	G	G	G	G	A	C	d	d	d	d	d	d	d	d	d	d	d	d	d	d	C	**A**	C
4.3 ceramensis	T	A	A	C	**A**	C	G	**C**	C	T	A	T	**T**	C	**G**	**A**	**A**	G	G	**G**	C	d	d	d	d	d	d	d	d	d	d	d	d	d	d	C	G	T
4.4a tumulosa a	T	A	**G**	C	G	C	G	G	**T**	T	A	T	C	**Y**	A	G	G	G	G	A	C	d	d	d	d	d	d	d	d	d	d	d	d	d	d	**T**	G	T
4.4b tumulosa b	T	A	**G**	C	G	C	G	G	**T**	T	A	T	C	C	A	G	G	G	G	A	C	d	d	d	d	d	d	d	d	d	d	d	d	d	d	**T**	G	T

mutS

Clade 5C	M144	M151	M224	M245	M248	M249	M285	M547	M597	M618	M621	M634	M690
5.1 daii/penghuensis	C	G	C	T	C	T	A	A	A	C	C	A	T
5.2a slieringsi a	C	G	C	T	C	T	A	A	**M**	C	C	A	T
5.2b slieringsi b	C	**R**	C	T	C	T	A	A	A	C	C	A	T
5.3 acuta/abrupta	C	G	C	T	C	T	A	A	A	C	Y	A	T
5.4 maxima	C	G	**T**	T	C	T	**C**	A	A	**T**	**T**	A	**C**
5.5 densa	C	G	C	T	**A**	T	A	**T**	A	C	C	A	T
5.6 wanannensis	C	G	C	T	C	**C**	A	A	A	C	**T**	A	T
5.7 exilis	**T**	G	C	T	C	T	A	A	A	C	C	A	T
5.8 lochmodes	C	G	C	**C**	C	T	A	**T**	A	C	C	**W**	T

28S

Clade 5C	S056	S060	S120	S155	S157	S164	S232	S292	S342	S386	S509	S531	S534	S544	S546
5.1 daii/penghuensis	G	G	**C**	**T**	**G**	**A**	**A**	**C**	G	G	C	**T**	C	C	**T**
5.2a slieringsi a	G	**T**	T	C	C	d	G	T	**A**	G	C	C	C	C	C
5.2b slieringsi b	G	**T**	T	C	C	d	G	T	**A**	G	C	C	C	C	C
5.3 acuta/abrupta	G	G	T	C	**G**	d	G	T	G	G	**A**	C	**G**	C	C
5.4 maxima	G	G	T	C	C	d	**A**	T	G	G	C	C	C	**G**	C
5.5 densa	G	G	T	C	**G**	d	G	T	G	G	**A**	C	C	C	C
5.6 wanannensis	K	G	T	C	C	d	R	T	G	**S**	C	C	C	C	C
5.7 exilis	**T**	G	T	C	C	d	G	T	G	G	C	C	C	C	C
5.8 lochmodes	G	G	T	C	**G**	d	G	T	G	G	**A**	**T**	C	C	C

Suppl. Fig. X: Character-based barcodes for clade 4 and 5C *Sinularia* based on *mtMutS* and 28S rDNA sequences. Species are numbered to reflect the clade to which they were assigned in RADseq species delimitation analyses. Shaded, bolded nucleotides are species-diagnostic characters; bolded IUPAC ambiguity codes indicate polymorphic positions at which the alternative allele is private to a species while those shown in yellow are simple (non-diagnostic) polymorphisms. Each string of characters represents a "pure diagnostic combination" (deSalle et al. 2005) unique to a species. Nucleotide positions are numbered from the start codon of mtMutS and from the conserved motif AAACCGTTG in 28S (approx. 40 bp downstream of forward PCR primer 28S-Far).
